# Cocaine Anæsthesia during Labour

**Published:** 1900-10-20

**Authors:** 


### COCAINE ANÆSTHESIA DURING LABOUR.

Dr. Marx, of New York,[1] publishes another series of cases in which he has produced anæsthesia to such a degree as to ensure practically painless labour by injecting cocaine into the spinal cord, a proceeding which he says is "a method ideally suited to mitigate or absolutely allay the dreadful pains of a normal labour, with no danger to the mother and none to the child, immediate or remote." There seems no doubt about the possibility of producing sensory paralysis from about the umbilicus downwards by this means, and some of the cases given are very striking. We must, however, draw attention to one or two points which come up even in a paper written by so strong an advocate of the operation as Dr. Marx. First of all we have the fact that it is not a question of a single operation which will carry right through the labour. The injection may have to be repeated, perhaps more than once, and in regard to this repetition it must be remembered that in describing the technique the author of the paper tells us that "the patient's back, from the coccyx to the middle of the dorsal vertebræ, is rendered absolutely sterile after the usual methods, even as the abdomen is sterilised before abdominal section," and that "asepsis of person, of instruments, of patient, of solution, is the only safeguard, and this must be as carefully attended to as if the belly were about to be opened. The operation should be done only by one trained in surgical asepsis, or else surely bad results will often occur in the form of septic intraspinal complications." The possibility of having to repeat an operation requiring all these precautions in the course of a labour seems a somewhat serious matter, and one which detracts considerably from the apparent simplicity of the proceeding. It is also to be remembered that one of the dangers of this method—which is said to be so free from danger to both mother and child—is "collapse from cocaine." Even in the simple cases "disagreeable features frequently occur," among them being nausea, vomiting, severe headache, profuse perspiration, chilly sensations, and temperatures up to 102° F. or 103° F.; and although it may be possible to control some of these "disagreeable features" by the administration of other alkaloids, they must not be overlooked in estimating the advantages and disadvantages of the method.

[1] Medical Record, Oct. 6th.

